# Investigation of Metabolic and Inflammatory Disorder in the Aging FGF21 Knockout Mouse

**DOI:** 10.1007/s10753-024-02032-3

**Published:** 2024-04-24

**Authors:** Lu-Qiong Cai, Xiu-Chun Li, Yang-Yue Wang, Yu-Xin Chen, Xia-Yan Zhu, Zi-Yi Zuo, Yi-Qun Si-Ma, Yi-Nuo Lin, Xiao-Kun Li, Xiao-Ying Huang

**Affiliations:** 1 Division of Pulmonary Medicine, the First Affiliated Hospital, Wenzhou Medical University, Wenzhou Key Laboratory of Interdiscipline and Translational Medicine, Wenzhou Key Laboratory of Heart and Lung, Wenzhou, Zhejiang 325000 China; 2 School of Pharmacy, Wenzhou Medical University, Chashan University Park, Wenzhou, Zhejiang 325000 People’s Republic of China

**Keywords:** aging, fibroblast growth factor 21, inflammatory response, fatty liver, transcriptomics, metabolomics and lipidomics

## Abstract

Aging is a physiological condition accomplished with persistent low-grade inflammation and metabolic disorders. FGF21 has been reported to act as a potent longevity determinant, involving inflammatory response and energy metabolism. In this study, we engineered aging FGF21 knockout mice of 36–40 weeks and observed that FGF21 deficiency manifests a spontaneous inflammatory response of lung and abnormal accumulation of lipids in liver. On one hand, inflamed state in lungs and increased circulating inflammatory cytokines were found in FGF21 knockout mice of 36–40 weeks. To evaluate the ability of FGF21 to suppress inflammation, a subsequent study found that FGF21 knockout aggravated LPS-induced pulmonary exudation and inflammatory infiltration in mice, while exogenous administration of FGF21 reversed these malignant phenotypes by enhancing microvascular endothelial junction. On the other hand, FGF21 knockout induces fatty liver in aging mice, characterized by excessive accumulation of triglycerides within hepatocytes. Further quantitative metabolomics and lipidomics analysis revealed perturbed metabolic profile in liver lacking FGF21, including disrupted glucose and lipids metabolism, glycerophospholipid metabolism, and amino acid metabolism. Taken together, this investigation reveals the protective role of FGF21 during aging by weakening the inflammatory response and balancing energy metabolism.

**Supplementary Information:**

The online version contains supplementary material available at 10.1007/s10753-024-02032-3.

## Introduction

The process of aging is a multifaceted and widespread occurrence that is shaped by a combination of environmental factors, stochastic events, genetic variations, and epigenetic changes across various cell types, tissues, and their intricate interplays throughout an individual's lifespan [1–3]. At the same time, aging always goes with persistent and low-grade inflammation as well as metabolic disorder, both of which are considered to take critical roles in age-related pathologies [4, 5]. Nevertheless, the precise etiology, particular pathological effect, and potential role in contributing to adverse conditions remain unknown.

Different from acute inflammatory process in response to harmful insults such as traumatic tissue injury or an invading pathogen, chronic inflammation during aging tends to be of low grade and persistence, leading to tissue degeneration finally. Possible mechanisms behind the special process includes (1) abnormal production of cytokines from activated immune cells and damaged nonimmune cells [6, 7]; (2) the modulation of “anabolic signaling”; for example, metabolic synthesis induce production of tumor necrosis factor-α and IL-6 to lower metabolic synthesis back [8, 9]. The contributions of above studies require further study and there is still less acknowledgement about the complete mechanisms and pathways.

Disturbed metabolic process (i.e., obesity, dyslipidemia, glucose intolerance, insulin resistance) are common during unhealthy aging, which are featured by ectopic fat accumulation and abnormal lipid metabolism. Although there is agreement that familial aggregation and cluster are notable characteristic of these conditions, much debate remains unsettled over its pathogenesis. Metabolic perturbances may closely involve in aging development as metabolic changes like increased glycolysis and disturbed lipid metabolism are present in senescence [10, 11]. In addition, lipid metabolism dysfunction caused by epigenetic changes may accelerates aging [12] and the impairment of genes involved in lipids synthesis extends lifespan.

Fibroblast growth factor 21 (FGF21) initially attract interests as a great metabolic regulator of glucose and lipids metabolism. As evidence shown, FGF21 is reported to prevent fatty liver formation and insulin resistance in obese mice model and numerous studies showed FGF21 analogue reduced fat mass and alleviated hyperglycaemia, insulin resistance, dyslipidaemia in NASH [13, 14]. Furthermore, when subjected to a ketogenic diet, mice lacking FGF21 (FGF21 KO mice) exhibited significant fatty liver accumulation and compromised fatty acid oxidation [15]. With the deepening of the research, FGF21 was reported to possess the excellent abilities of anti-inflammation [16–18]. Although the role of FGF21 in the regulation of inflammatory process and metabolic process has been well elucidated in previous studies, the effect of FGF21 deficiency on aging remain unsettled. Regarding that disrupted metabolism and sterile inflammation are significant phenotype of aging, hypothesis was put forward that FGF21 may take a critical role in aging as most if not all aging-involved diseases share an inflammatory and disturbed-metabolic signature. Previous study shows that circulating levels of FGF21 increase with aging in both rodents and humans [19]. Though report have been made in clarifying the altered level of FGF21 in aging, the targeted organs, histological changes and pathological mechanism still need exploration.

After genomics, transcriptomics and proteomics, metabolomics emerged as a new approach to characterize metabolic profiling of biological processes [20–22]. When an individual life is in response to physiological stimuli as well as pathological insults, or influenced by genetic or environmental factors, the metabolic profile changed. Although the FGF21 is a key metabolic-regulatory factors, the reports about specific metabolic events of disease pathogenesis and progression remain limited. To fill in the gap, a targeted metabolomics profiling and enriched pathway analysis on liver samples was performed to identify hepatic metabolic alterations and investigate the potential effect induced by FGF21 deletion. Exploring metabolic changes enables the screening of potential biomarkers or therapeutic targets in FGF21-lacking conditions [23]. Therefore, our study not only deepens our comprehension of the role of FGF21 during aging but provides inflammatory and metabolic mechanism underlying it.

## Result

### FGF21 Knockout Induces Inflammatory Response of Lung and Steatohepatitis Spontaneously in 36–40 Weeks Mice

**Fig. 1:**
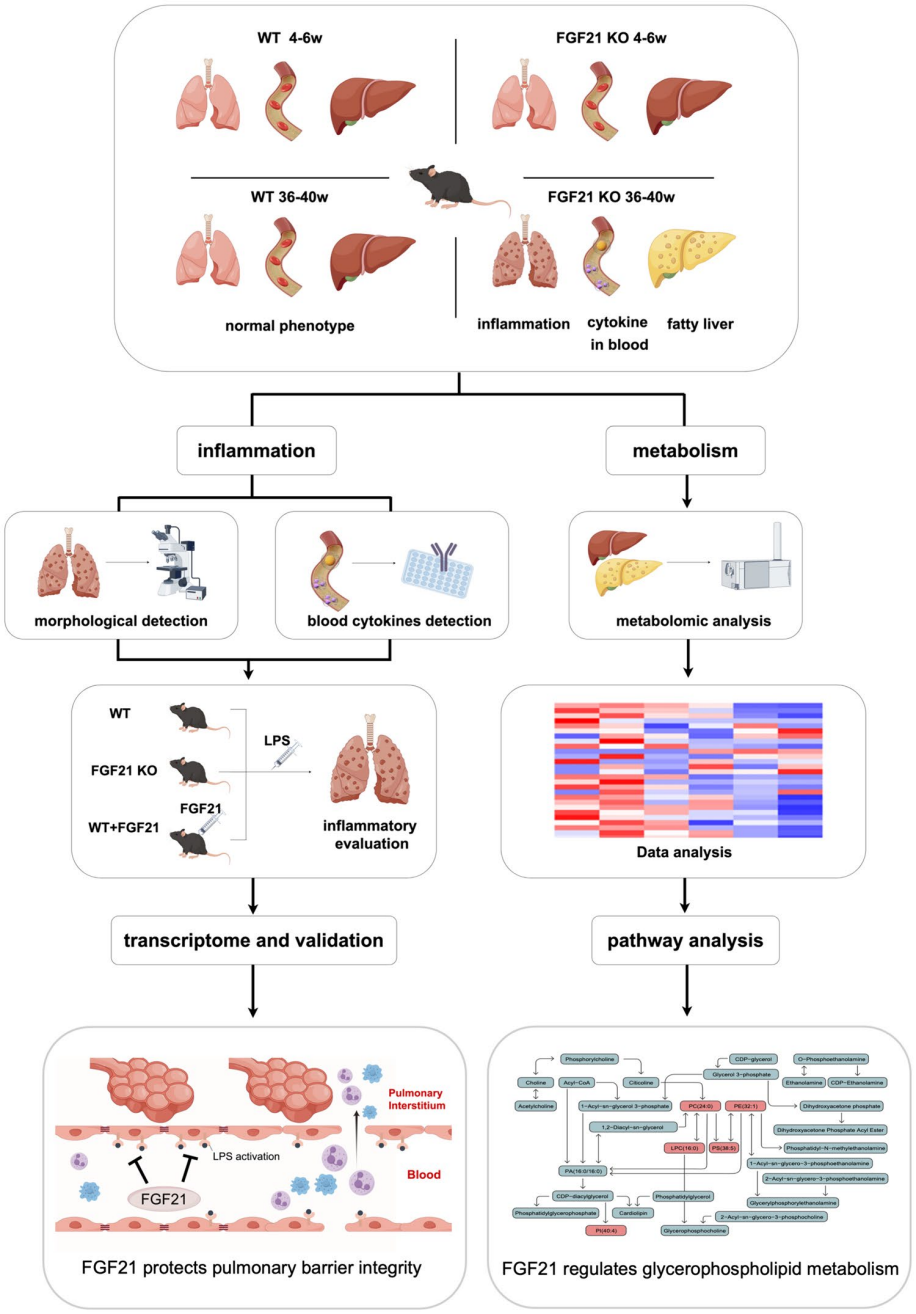
Graphical abstract: FGF21 regulate inflammatory response and energy metabolism during aging.

**Fig. 2:**
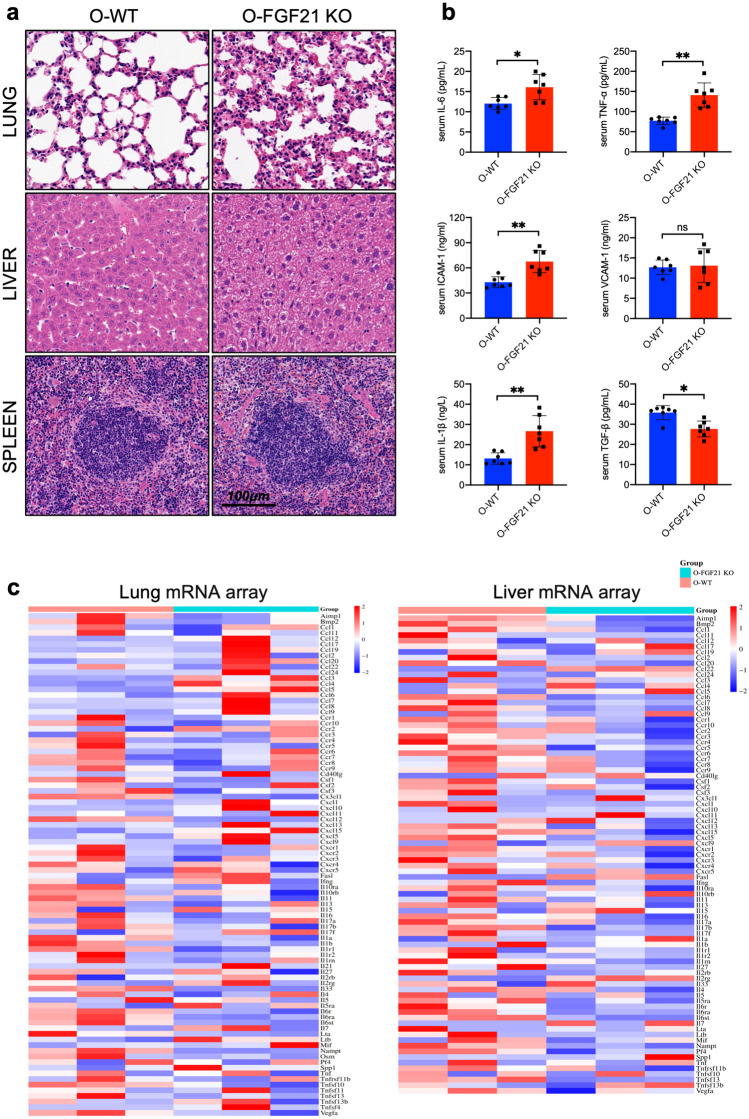
FGF21 knockout induces inflammatory response of lung and fatty liver spontaneously in 36–40 weeks mice. **a** Representative H&E‐stained sections of lungs, livers and spleens in 36–40 weeks mice of FGF21 KO mice and WT mice, ×200, Scale bar, 100 μm (n = 7). **b** The cytokines concentrations in serum of mice aged at 36–40 weeks (n = 7; ^*^p < 0.05, ^**^p < 0.01 vs. O-WT mice). **c** The heatmap summarize the mRNA level of inflammatory cytokines and related receptors in the liver and lung of O-FGF21 KO mice and O-WT mice. Gene expression levels were normalized as Log2(fold change in O-FGF21 KO mice vs O-WT mice) (blue: downregulated, red: upregulated, white: medium change) (n = 3). O-WT and O-FGF21 KO means mice aged at 36–40 weeks.

The positive role of FGF21 against inflammatory progression and metabolic disorder has been demonstrated in previous study. Another unexpected observation that these FGF21 knockout mice developed spontaneous inflammatory response of lung and fatty liver as well as increased serum cytokines in 36–40 weeks mice (Fig. 1). The morphology of lung, liver, and spleen were identified to evaluate its condition (Fig. 2a). These structures showed no difference between those two groups at age of 4–6 weeks (Supplementary Fig. 1a). When mice grow to 36–40 weeks, the lung showed thickened interstitium and more inflammatory cell infiltrations in FGF21 KO mice (Fig. 2a). In liver, FGF21 knockout lead to visible change like fatty liver such as hepatocyte ballooning steatosis and mixed inflammatory infiltration. No significant disruption was seen in the architecture of the spleen in FGF21 knockout mice as no dramatic expansion of immune cells is typically observed. Serum cytokines such as IL-6, TNF-α, IL-1β, and ICAM-1 were higher in FGF21 KO mice aged at 36–40 weeks mice. And VCAM-1 trend upward without significant difference. In addition, cytokine TGF-β with anti-inflammation effect is downregulated (Fig. 2b). Differently, IL-6, TNF-α, IL-1β, ICAM-1 and VCAM-1 showed no significant difference between two groups of mice aged at 4–6 weeks (Supplementary Fig. 1b). But TGF-β is upregulated in FGF21 KO mice of 4–6 weeks compared with control mice of 4–6 weeks.

The alterations observed above have been linked to multiple processes of immune activation and inflammation. To get a comprehensive list of inflammatory genes that are changed when FGF21 is absent, we performed PCR array on inflammatory genes. As the heatmap shown, the deletion of FGF21 perturbed the pulmonary, hepatic and splenic expression pattern of inflammatory genes. The significantly differential genes were selected with fold regulation greater than 2 and *P*-value < 0.05. A total of 3 genes were remarkably upregulated in the lungs of FGF21 KO mice, FGF21 knockdown dramatically upregulated gene expression of chemokines (Ccl3, Ccl4 and Ccl5) in lung (Fig. 2c). And hepatic expression of Il15 was also upregulated. Two genes were upregulated in spleen, including interleukins Il5ra and Cxcr5 (Supplementary Fig. 1c). These gene expression profiles demonstrated that absence of FGF21 increases part of gene expression of chemokines class and interleukin class, with the notable highlight of upregulation in the lung.

The downregulating inflammatory genes expressed in the absence of FGF21 in the lungs, livers, and spleens of FGF21 KO mice also was evaluated. In the lung, 9 genes were downregulated in FGF21 KO mice, including Cx3cl1, Cxcl12, Il11, Il1a, Il33, Il6r, Il6ra, Il6st and Nampt (Fig. 2c). Meanwhile, those negatively regulated genes were found in the liver, including Bmp2, Ccl20, Ccl8, Ccr6, Cxcl5, Cxcr1, Il17b, Il5ra, Il6ra, Il6st, Nampt, Pf4 and Tnfsf13. Meanwhile Cxcl9 was also downregulated in the spleen (Supplementary Fig. 1c). In brief, FGF21 knockout lead to systemic inflammation, abnormal accumulation of fat in liver and sterile inflammatory response of lung.

### Knockout of FGF21 Aggravates LPS-induced Permeability and Inflammatory Infiltration in Mice

Following above result that FGF21 genetic deletion led to chronic sterile inflammation of lung related with aging, the role of FGF21 in exogenous inflammatory response of lung was studied. LPS is a lipoglycan found on the outer membrane of Gram-negative bacteria and functions as an activator of inflammatory response. A murine model of intratracheal LPS administration was regarded as an established model to induce lung inflammation. After 24 h, lungs were either fixed and subjected to histopathological analysis for lung injury, or lavaged to collect BALF. It’s known that estriol reduces pulmonary immune cell recruitment and inflammation to protect female mice from respiratory infection [24]. And FGF21 knockout has non-negligible impact on inflammatory response of lung of aging mice. Consequently, only 4–6 weeks male mice were utilized to exclude the special side effect from female mice in this study.

**Fig. 3:**
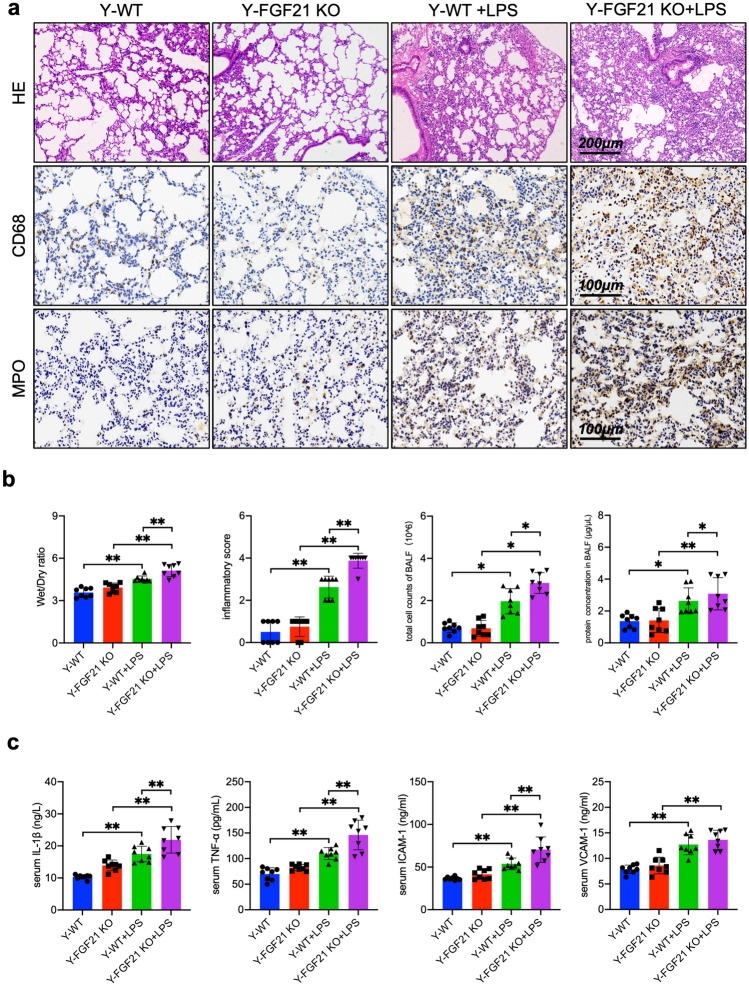
Knockout of FGF21 aggravates LPS-induced permeability and inflammatory infiltration in mice. **a** Representative H&E‐stained lung sections (×100, Scale bar, 200 μm) and images of lung sections stained with anti-CD68 antibody and anti-MPO antibody respectively (×200, Scale bar, 100 μm). **b** Pathological inflammatory scores, Wet/Dry ratio of lungs, BALF total cell counts and BALF protein concentration (n = 8). **c** The serum level of ICAM-1 (ng/ml), TNF-α (ng/L), VCAM-1 (ng/ml) and IL-β (ng/L) (n = 8). Quantitative data are presented as mean ± SD and compared by one-way ANOVA tests (^*^P < 0.05, ^**^P < 0.01). Y-WT and Y-FGF21 KO means mice aged at 4–6 weeks.

Young WT mice and FGF21 KO mice were intratracheally administrated with same level of LPS (10 mg/kg, i.t.). After 24 h, H&E staining showed thickened alveolar walls in Y-WT mice with intratracheal injection of LPS. However, alveolar walls were more markedly thickened in Y-FGF21 KO+LPS mice (Fig. 3a). Lung tissue section immunohistochemistry staining reveal an increased CD68 and MPO positive cells amount in Y-FGF21 KO+LPS mice, which indicated a potent inflammatory infiltration. Inflammatory score was also higher in Y-FGF21 KO+LPS group compared to Y-WT+LPS group (Fig. 3b). In addition, knockout of FGF21 substantially increased Wet/Dry ratio of lung under LPS exposure, indicating more exudation in Y-FGF21 KO+LPS group (Fig. 3b). The total cell counts and protein concentration of BALF of Y-FGF21 KO+LPS mice were also higher (Fig. 3b). The serum level of ICAM-1, VCAM-1, TNFα, and IL-1β from all groups were measured to further assess systemic inflammation. The LPS-induced expression of ICAM-1, TNFα and IL-1β were dramatically elevated in Y-FGF21 KO ALI mice except for VCAM-1 (Fig. 3c). Collectively, these results indicate that FGF21 may also play a pivotal role in lung inflammation by regulating pulmonary permeability and inflammatory infiltration.

### FGF21 Administration Alleviates LPS-induced Exudation and Inflammatory Infiltration in Mice

**Fig. 4:**
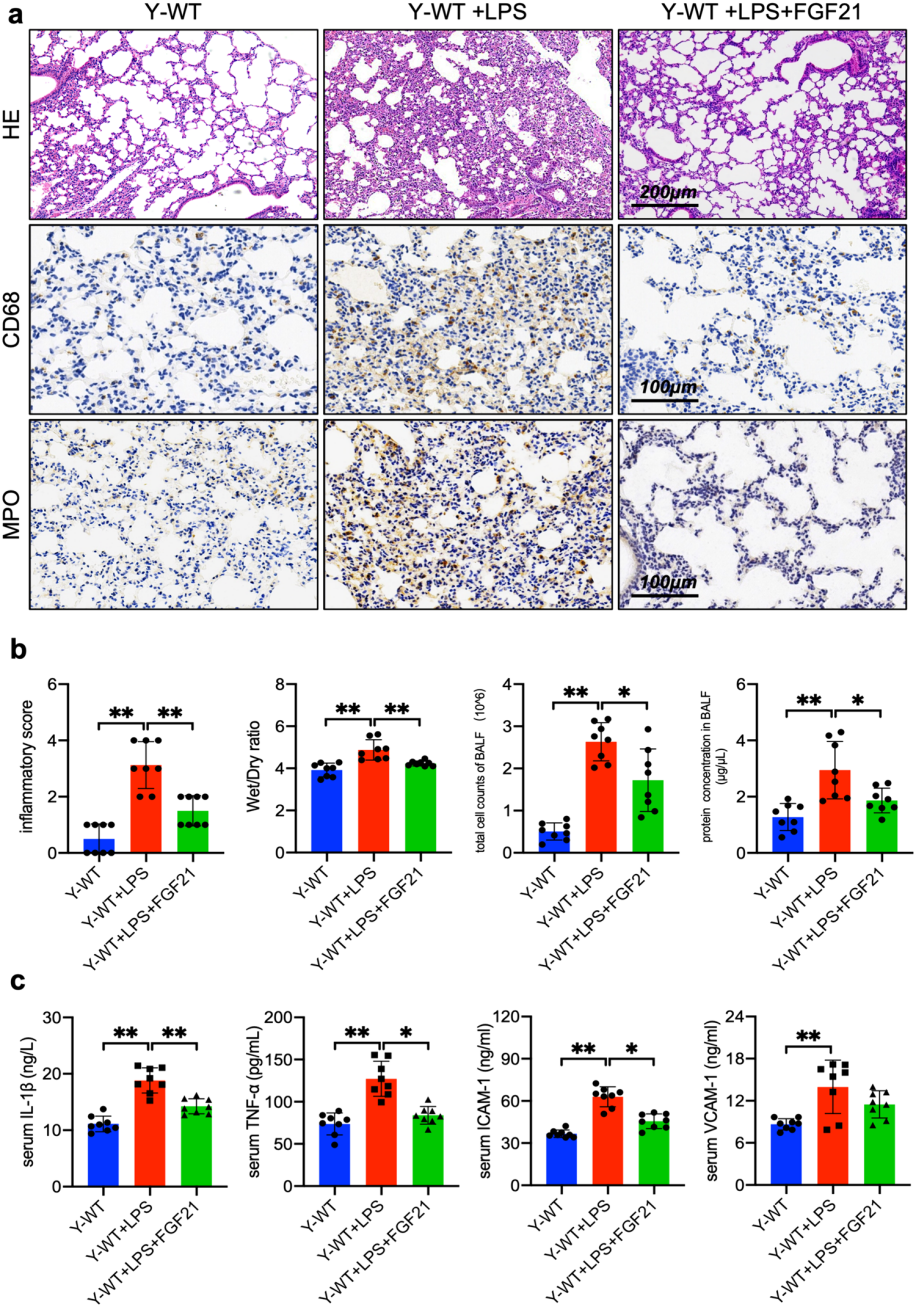
FGF21 administration alleviates exudation and inflammatory infiltration in LPS-induced ALI mice. **a** Representative H&E‐stained lung sections (×100, Scale bar, 200 μm) and images of lung sections stained with anti-CD68 antibody and anti-MPO antibody respectively (×200, Scale bar, 100 μm). **b** Pathological inflammatory scores, Wet/Dry ratio of lungs, BALF total cell counts and BALF protein concentration (n = 8). **c** The serum levels of TNF-α (ng/L), ICAM-1 (ng/ml), VCAM-1 (ng/ml) and IL-β (ng/L) (n = 8). Data are expressed as means ± SD and compared by one-way ANOVA tests. Quantitative data are presented as mean ± SD (^*^P < 0.05, ^**^P < 0.01). Y-WT and Y-FGF21 KO means mice aged at 4–6 weeks.

Given the exacerbation of pulmonary permeability and systemic inflammation caused by the knockdown of FGF21, this investigation sought to determine whether FGF21 administration could effectively alleviate LPS-induced pulmonary inflammation. Mice were intraperitoneally injected with recombinant human FGF21 (1.5 mg/kg, i.p.) half an hour after LPS injection. Lungs, BALF and serum of all groups were collected 24 h later to evaluate pulmonary permeability and the inflammatory situation mentioned above. H&E staining showed FGF21 administration alleviated interstitial thickening and the collapse of alveolus (Fig. 4a). The immunohistochemistry showed less inflammatory infiltration in LPS-injected mice administrated with FGF21. FGF21 administration lowered inflammatory score as well (Fig. 4b). In addition, FGF21 administration decreased lung Wet/Dry ratio, total cell counts and protein concentration of BALF. These results further suggest that FGF21 could attenuate leakage and inflammatory infiltration in LPS-induced pulmonary inflammation. Furthermore, the LPS-stimulated serum expressions of ICAM-1, TNFα and IL-1β were reduced by FGF21 administration (Fig. 4c). In conclusion, FGF21 administration alleviates LPS-induced exudation and inflammation in murine lung.

### The Effect of FGF21 on Inflammatory Response of Lung is Related to Tight Junctions in Endothelium

**Fig. 5:**
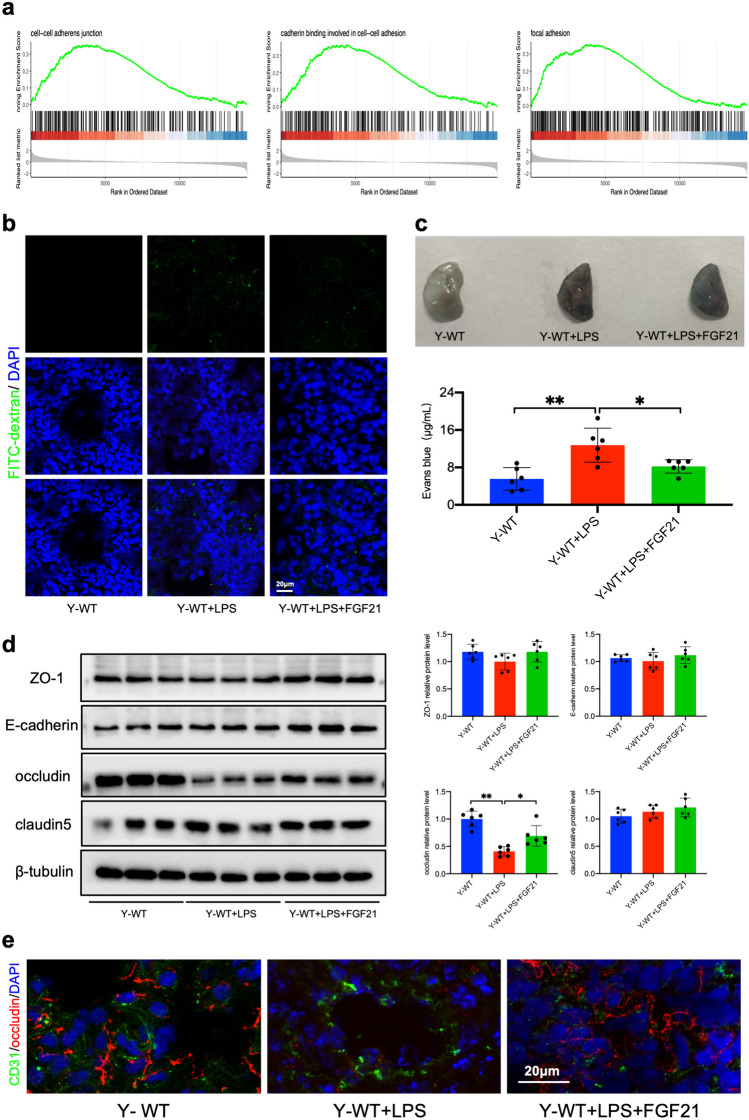
FGF21 administration attenuates endothelial barrier dysfunction *in vivo*. **a** GSEA enrichment analysis of differential expressed genes. **b** Confocal laser scanning microscopy images of lung with FITC-dextran extravasation. Scale bar, 20 μm. **c** Lung appearance after Evans blue dye injection and Evans blue permeability assay (n = 6). **d** Expression of ZO-1, E-cadherin, caludin5 and occludin were measured by western blot and analyzed by Image J (n = 6). Quantitative data are presented as mean ± SD (^*^P < 0.05; ^**^P < 0.01) Y-WT and Y-FGF21 KO means young mice aged at 4–6 weeks. **e** Merge images of confocal laser scanning microscopy of lung immunofluorescence section showing CD31 (green), occludin (red), DAPI (blue). Scale bar, 20 μm.

To gain further insight into the full mechanisms of FGF21 on inflammatory response, RNA sequencing was used to investigated how FGF21 affected the lung transcriptome. The gene expression profile of murine lung with or without FGF21 deficiency was analyzed. 693 different expressed genes (DEGs) including 461 upregulated genes and 232 downregulated genes were obtained (Supplementary Fig. 2a). Gene set enrichment analysis (GSEA) revealed a specific enrichment of genes in biological processes about cell-cell adherens junction, cadherin binding involved in cell-cell adhesion, and focal adhesion (Fig. 5a). The cellular junctions mentioned above were important parts in endothelial barrier integrity, which controls the transport of various molecules and restricts permeability. Tight junction proteins especially occludin, claudin5, ZO-1 and E-cadherin tightly connect intercellular gaps and play a critical role in endothelial barrier integrity. The above results showed that as hypothesized FGF21 could improve pulmonary barrier integrity and enhance tight junction proteins.

FITC-dextran pulmonary permeability analysis and Evans blue extravasation assessment were employed to evaluate the effect of FGF21 on endothelial leakage in LPS-induced pulmonary inflammation. As expected, the FITC-dextran leakage and Evans blue extravasation were reduced by FGF21 administration in mice underwent LPS treatment (Fig. 5b and c). Considering the pulmonary endothelial barrier is associated with tight junction proteins closely, the expressions of related proteins in lung were also evaluated by western blot. As a result, occludin was decreased after LPS injection while ZO-1, E-cadherin and claudin5 showed no significant downregulation. And FGF21 administration reversed the expression of occludin (Fig. 5d). Immunofluorescence of occludin also showed the same effect of FGF21 on occludin expression (Fig. 5e). In summary, FGF21 could improve endothelial barrier integrity and enhance tight junction protein occludin *in vivo*, suggesting the mechanisms underlying the protective role of FGF21 against inflammatory response of lung.

### Transfection of FGF21 Attenuates LPS-induced Endothelial Barrier Disruption and Enhance Tight Junctions *In Vitro*

**Fig. 6:**
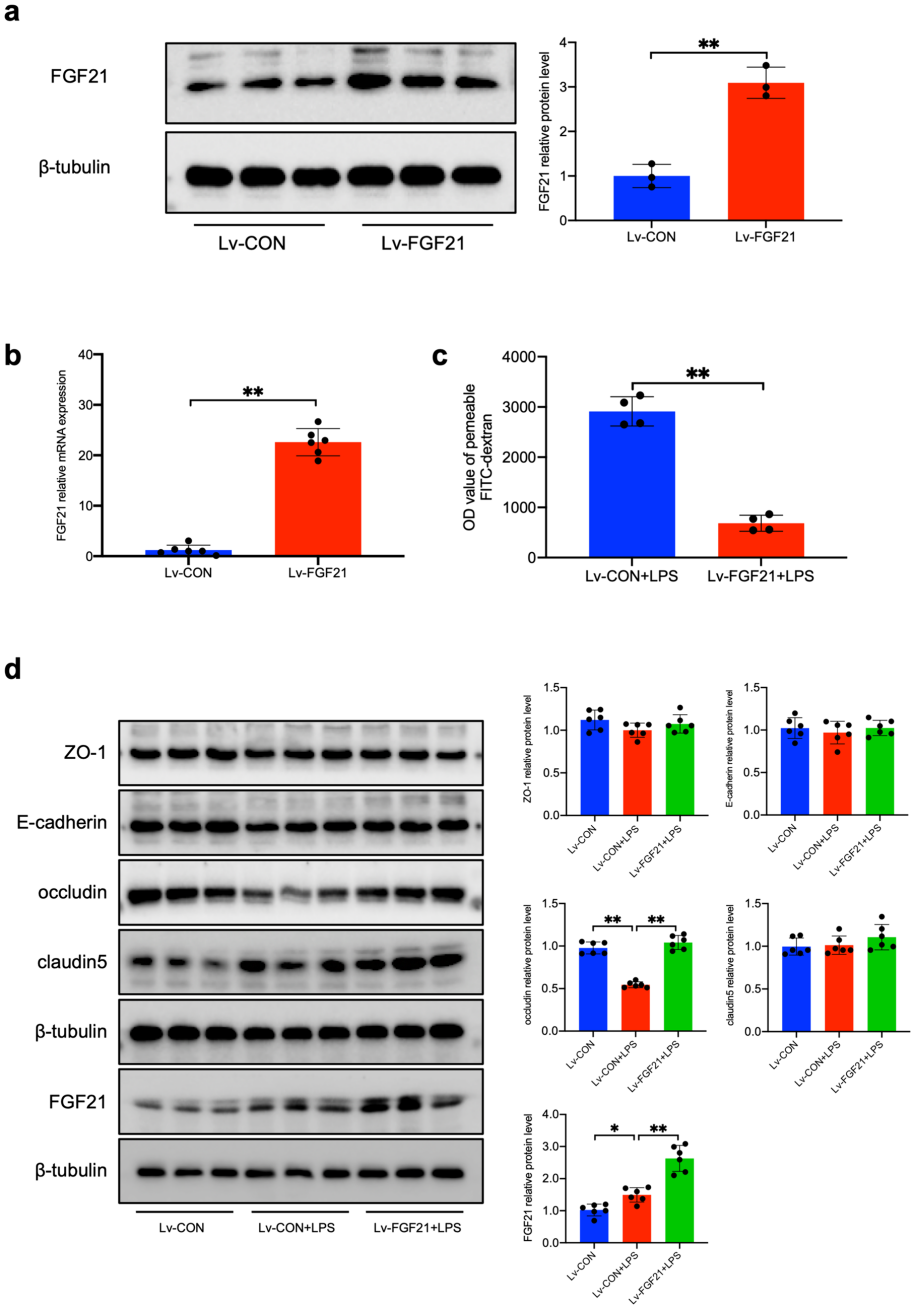
Transfection of FGF21 rescues endothelial barrier dysfunction *in vitro*. **a** The protein expression of FGF21 (n = 3). **b** The mRNA expression of FGF21 (n = 6). **c** The relative OD value of leaking FITC-dextran (n = 4). **d** The expression of ZO-1, E-cadherin, caludin5, occludin and FGF21 were measured by Western blot and analyzed by Image J (n = 6). Quantitative data are presented as mean ± SD (^*^P < 0.05 and ^**^P < 0.01).

Then the effect of FGF21 on barrier function *in vitro was explored.* HUVEC cell lines were transfected with recombinant lentivirus expressing mouse FGF21 (Lv-FGF21) or vector control (Lv-CON) (Fig. 6a and b). The protein expression level of FGF21 in Lv-FGF21 was almost threefold increase compared with Lv-CON (Fig. 6a). The mRNA level of FGF21 was also upregulated (Fig. 6b). FITC-dextran monolayer permeability analysis was performed to evaluate the effect of FGF21 on monolayer barrier function. Result showed that leaking FITC-dextran (70 KD) caused by 24 h LPS stimulation was significantly less in Lv-FGF21+LPS (Fig. 6c) than that of Lv-CON+LPS. Western Blot quantification showed that lentivirus transfection enhanced FGF21 stably under LPS stimulation (Fig. 6d). In addition, occludin decreased significantly 24 h after LPS exposure and FGF21 overexpression enhanced the occludin expression (Fig. 6d). Taken together, this study suggested that FGF21 overexpression could enhance LPS-induced endothelial monolayer barrier integrity and increase the occludin expression *in vitro*.

### The Effect of FGF21 on Endothelial Barrier Function is Related to JNK MAPK Signaling Pathway

**Fig. 7:**
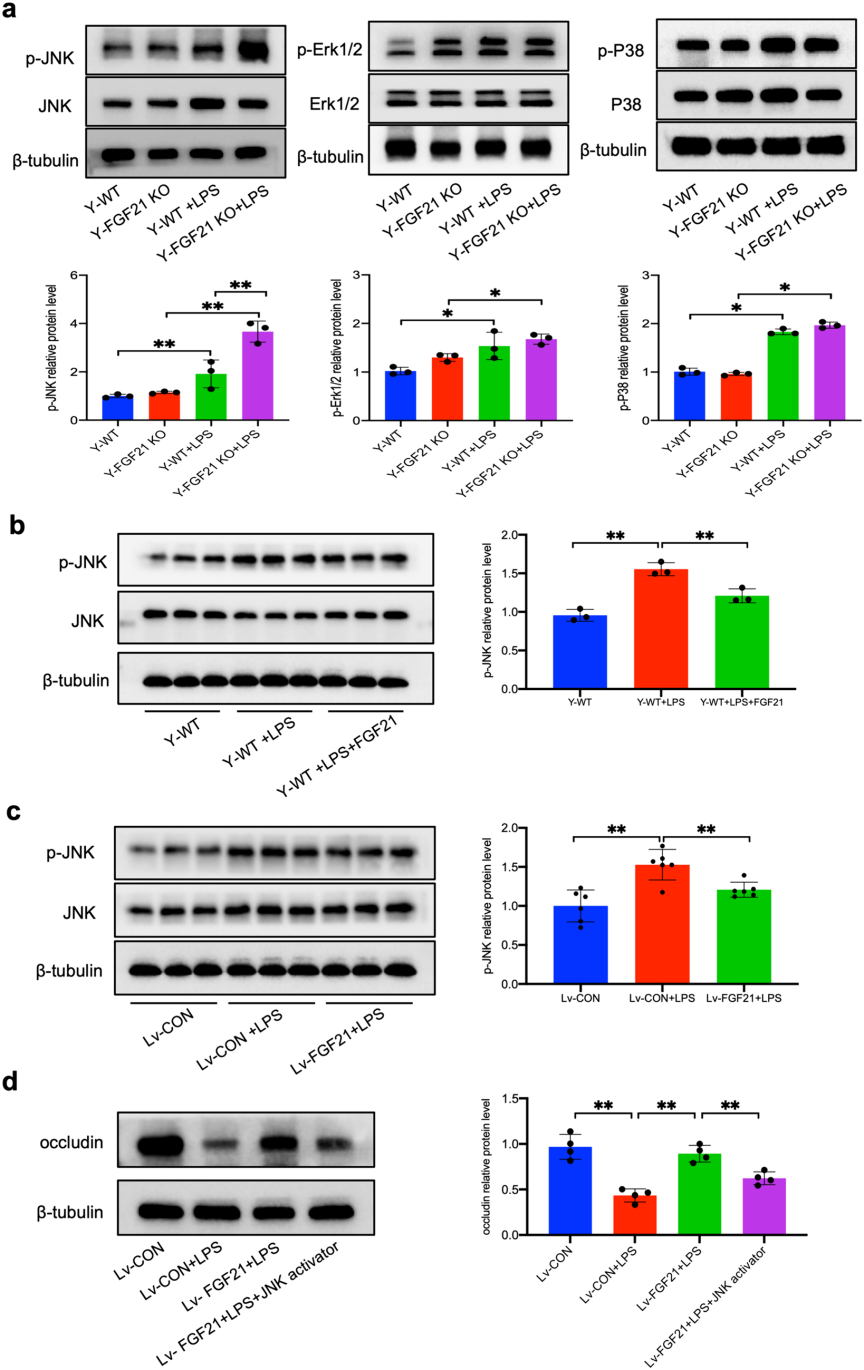
FGF21 improves occludin expression via JNK MAPK signaling pathway. **a** The expression of p-JNK, JNK, p-Erk1/2, Erk1/2, p-P38 and P38 were measured by Western blot. Image J was used to quantify, and β-tubulin was used as loading and normalization control (n = 3; ^*^P < 0.05, ^**^P < 0.01). **b** The expression of p-JNK and JNK measured by Western blot (n = 3; ^*^P < 0.05, ^**^P < 0.01). **c** The expression of p-JNK and JNK measured by Western blot (n = 6; ^*^P < 0.05 and ^**^P < 0.01). **d** The expression of occludin measured by Western blot. (n = 4; ^*^P < 0.05, ^**^P < 0.01).

To elucidate the underlying signaling mechanisms, Kyoto Encyclopedia of Genes and Genomes (KEGG) pathway enrichment analysis was further performed. KEGG enriched analysis indicated that the protective effect of FGF21 may be related to cytokine-cytokine receptor interaction and MAPK signaling pathway (Supplementary Fig. 2b). JNK MAPK signaling pathway is a crucial signaling pathway via which LPS and pro-inflammatory cytokines disturb endothelial barrier function [25]. In murine model, the phosphorylation level of MAPK was elevated in Y-WT+LPS compared with Y-WT. While simply JNK MAPK was significantly upregulated in Y-FGF21 KO+LPS when compared with Y-WT+LPS, indicating the potential role in protecting barrier integrity. The phosphorylation of Erk1/2 and P38 showed no changes between two groups (Fig. 7a). FGF21 administration inhibited activation of JNK MAPK both *in vivo* and *in vitro* as shown by immunoblots (Fig. 7b and c). And the rescue of FGF21 on occludin downregulation is reversed by JNK activator Anisomycin with a dose of 1 μg/mL (Fig. 7d). Taken together, the protective effect of FGF21 on endothelial barrier function is related to suppression of JNK MAPK signaling pathway.

### Metabolic and Lipidomic Profiling Analysis of the Liver of FGF21 Knockout Mice

**Fig. 8:**
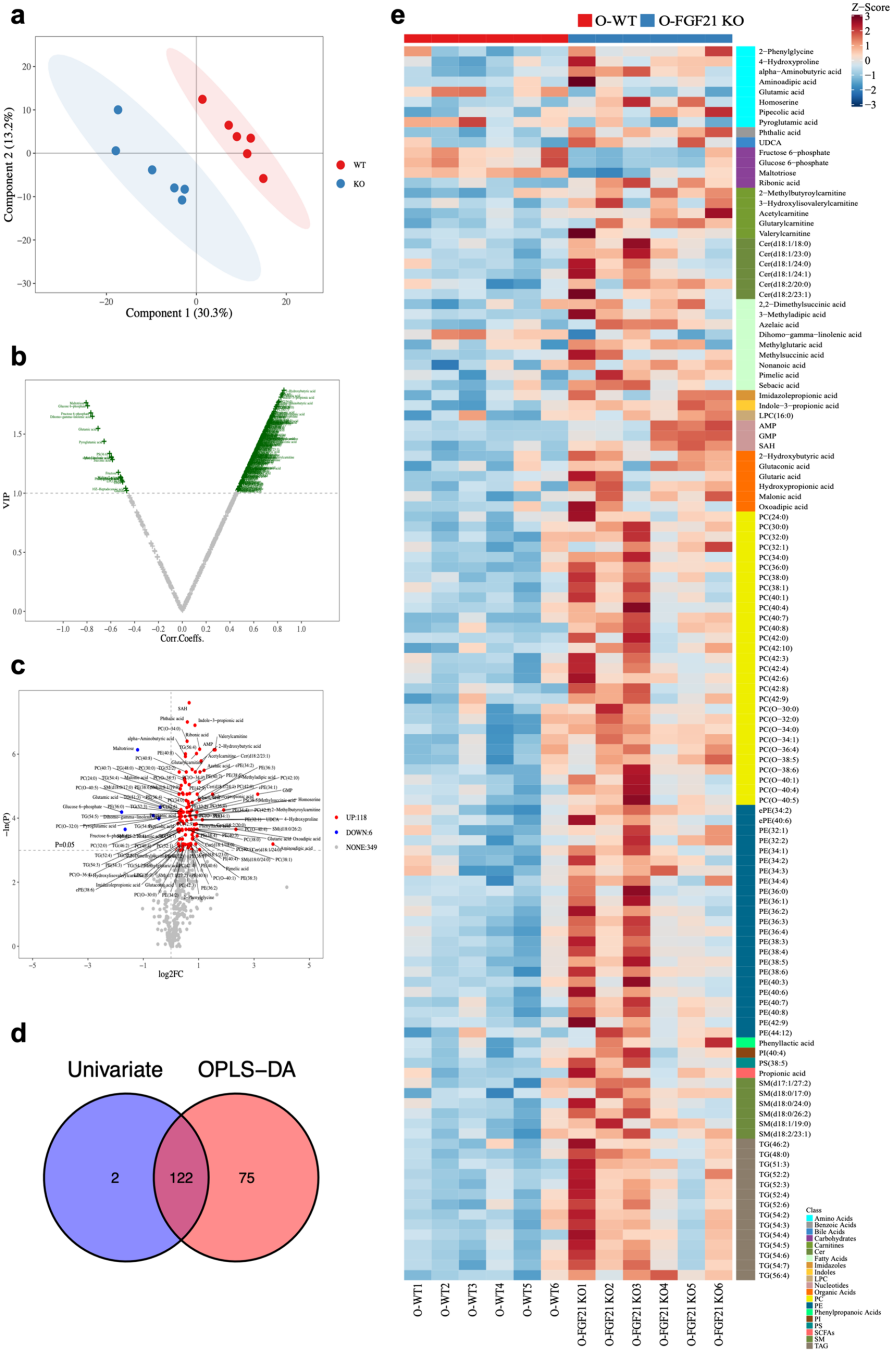
The metabolome profile of livers obtained from the FGF21 KO mice. **a** OPLS-DA is performed on the data of liver samples from O-FGF21 KO mice and O-WT mice. **b** Volcano plot is utilized to screen reliable potential biomarkers based on OPLS-DA model. **c** Volcano plot shows differential metabolites generated by univariate analysis. **d** Venn diagrams of differential metabolite were obtained using intersection between univariate analysis and OPLS-DA. **e** heatmap of differential metabolites between O-FGF21-KO mice and O-Wide-type mice.

As FGF21 knockdown led to fatty liver in aging mice, we further performed metabolomics to identify metabolic alteration in the liver, which is a major organ for metabolic balance. PCA, an unsupervised method, is applied as the first step in the separation procedure to reduce the dimension of data and make the observation more straightforward. Score plot with aggregated QC samples (QC samples gather together) indicates that the quality control is good, and the detection process is stable (Supplementary Fig. 2c). The overview principal component analysis (PCA) score plot showed that the FGF21 KO group was not clearly separated from the WT group. OPLS-DA, a supervised method that has a similar principle to PCA, is used to enhance the classification performance. OPLS-DA displayed a clearly separated cluster between the two groups (Fig. 8a). Based on OPLS-DA model, volcano Plot is helpful to select differential (statistical significantly) changed metabolites (Fig. 8b). Then by using univariate statistical analysis, the differential metabolites can also be obtained (threshold value for differential metabolites selection is: (1) P < 0.05 (2) |log2FC|> = 0, student T-test or Mann-Whitney U test, depending on the normality of data and homogeneity of variance) (Fig. 8c).

Differential metabolites by getting intersection from univariate statistics and multi-dimensional statistics, we can find 122 potential biomarkers which may play critical roles in biological processes related to the study (Fig. 8d and e). Among 122 differential metabolites, glucose 6-phosphate, fructose 6-phosphate, maltotriose, dihomo-gamma-linolenic acid, pyroglutamic acid, and glutamic acid are downregulated and the rest metabolites are upregulated. Venn Plot of differential metabolites from multi-dimensional statistics and univariate statistics is shown (Fig. 8d). Differential metabolites in the intersection set are more reliable, since they fit both criteria for selection.

All 122 measured metabolites are presented stratified by group in Supplementary Table 1. With targeted MS, 122 different endogenous metabolites were quantified including Amino Acids (n = 8), Carbohydrates (n = 4), Carnitines (n = 5), Cer (n = 6), Fatty Acids (n = 9), Nucleotides (n = 3), Organic Acids (n = 6), PC (n = 29), PE (n = 23), SM (n = 6), TAG (n = 14), Others (n = 9). As heatmap shown, most part of TAG as well as fatty acid are unregulated in FGF21 KO mice, which is consistent with the metabolic feature of fatty liver (Fig. 8e). And Carbohydrates such as Maltotriose, Fructose 6-phosphate, Glucose 6-phosphate are downregulated in KO mice, while Ribonic acid is upregulated. In addition, Aminoadipic acid and 2-Methylbutyroylcarnitine are upregulated in liver lacking FGF21 (Supplementary Fig. 3). The above selected biomarkers will be used in the following analysis for Pathway Enrichment analysis and Pathway Analysis.

### The Metabolic Pathway Enriched for FGF21’s Influence on Hepatic Metabolism

**Fig. 9:**
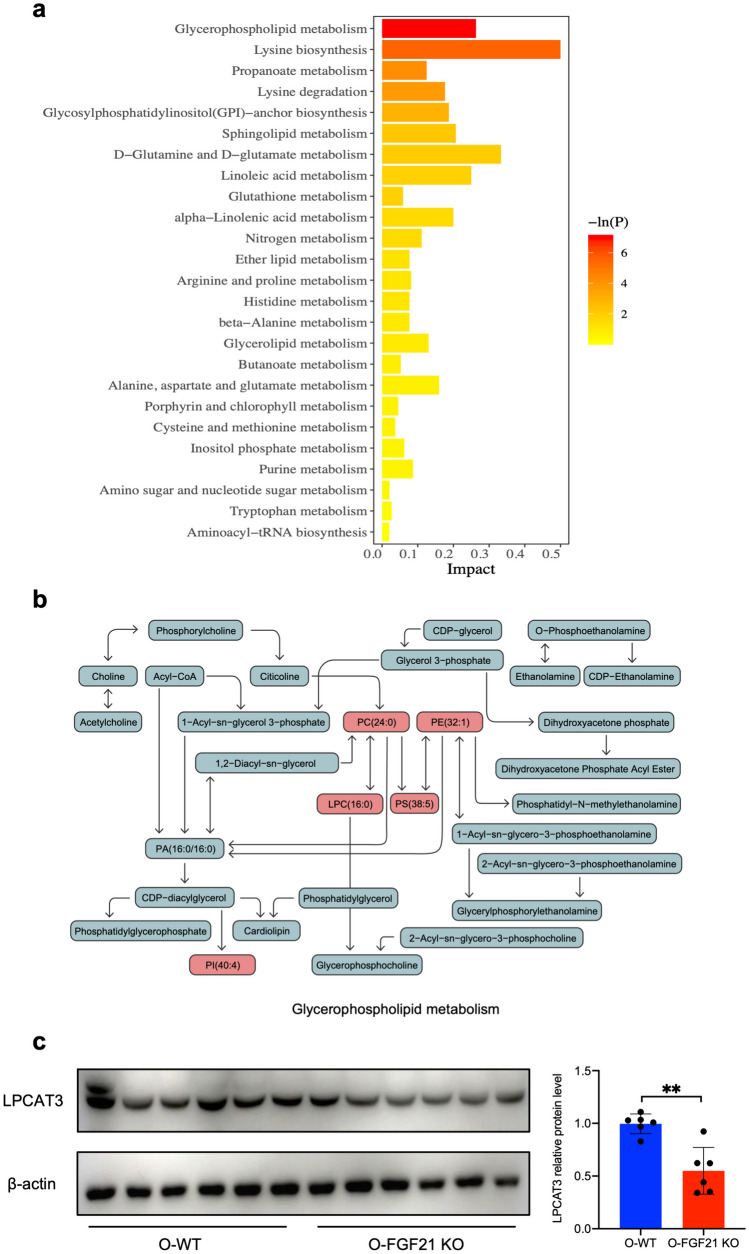
KEGG pathway enrichment analysis for metabolomics. **a** Pathway analysis of differential metabolites between hepatic tissues from O-FGF21 KO and O-WT mice (n = 6). **b** Metabolic pathway changes in liver between O-FGF21 KO mice and O-WT mice. The red shades represent significantly increased levels of metabolites. **c** Expression of LPCAT3 in liver was measured by western blot and analyzed by Image J (n = 6; ^**^P < 0.01).

Next, we linked the different expressed metabolites with metabolic pathways using the KEGG database. KEGG pathway analysis using these metabolites revealed the top enriched metabolic pathway (*p* < 0.005, impact > 0.1) was Glycerophospholipid metabolism (Fig. 9a). The changed metabolites of specific pathway are indicated in Fig. 9b. In FGF21 KO mice, LPCs show a trend towards increased levels with or without statistical significance (Supplementary Fig. 3). Lysophosphatidylcholine acyltransferase 3 (LPCAT3), converting LPC back to PC, was significantly downregulated in livers lacking FGF21, which indicated the possibility that FGF21 may regulate the Glycerophospholipid metabolism via LPCAT3 (Fig. 9c). Concluded to above results, FGF21 knockout lead to perturbed glucose and lipids metabolism in murine liver and maybe be involved in Glycerophospholipid metabolism.

## Discussion

Fibroblast growth factor 21 (FGF21) is an endocrine member of the FGFs family. Huge numbers of studies have revealed that FGF21 treatment can alleviate many age-related metabolic disorders [26] and exhibit anti-inflammatory property in multiple diseases like obesity [27], bacterial infection [28] and neurodegeneration [29]. Those aging-related phenotypes is always accompanied with metabolic dysfunction and long-term inflammatory condition, which may be a potential factor for cell senescence. Consequently, we wondered how FGF21 takes a role in aging process under normal balanced lifestyle.

FGF21 knockout mice and littermate control mice were kept under the same feeding and environmental conditions. This study unprecedentedly reported that compared with littermate control mice, FGF21 KO mice aged at 36–40 weeks had elevated inflammatory cytokines in perivascular serum alongside with substantial pathological changes of organs such as spontaneous inflammatory response in lung and abnormal accumulation of fat in liver. But the phenotypes mentioned above are not observed in young mice of 4–6 weeks between two groups, which indicated that it takes long time for FGF21 to take a role in aging. Above results may account for the inflammatory conditions and metabolic disorder during aging from genetic perspectives as well.

Other results also support that FGF21 take an anti-inflammatory role in degradative diseases [30]. One study has showed that FGF21 attenuates neurodegeneration by reducing neuroinflammation and oxidant stress through regulating the NF-κB pathway and AMPKα/AKT pathway, which enhances the protective effect on mitochondria in neurons [29]. Another report identified the conclusion that FGF21 is essential to counteract the renal inflammation during aging on a low-protein diet [31]. Those results make similar conclusion that FGF21 inhibited inflammation in aging-related diseases, which is consistent with our study. We further figure out whether exactly lack of FGF21 results in chronic and long-term inflammatory response in murine model. Regarding the limitation that the half-life of FGF21 is short and aging model is chronic, we further investigated the ability of FGF21 to respond to external inflammatory shock caused by LPS using FGF21-deletion mice and exogenous administration of FGF21. Result showed that FGF21 exert significantly anti-inflammatory properties, and the loss of FGF21 further aggravated the LPS-induced inflammatory phenotype. Our study deepens our understanding of FGF21 against inflammation in senescence.

Regarding the substantial inflammatory response of lung, transcriptome was further performed in this study to explore the mechanism of FGF21 on pulmonary inflammation. Result indicated that the enriched pathways were cell-cell adherens junction, cadherin binding involved in cell-cell adhesion, and focal adhesion, all of which was part of barrier integrity. Experiments on murine lung were made to measure the pulmonary permeability and results showed that FGF21 administration alleviated leakage caused by LPS-induced inflammation. Western blot of occludin also supports above conclusion. Recent findings also demonstrated an active role of FGF21 in barrier repairing, FGF21 was reported to decrease retinal permeability via regulating cellular tight junction, Chen *et al*. reported rhFGF21 preserved blood-brain barrier integrity through activation of PPARγ [17, 32–34]. Parallel investigation concluded that FGF21 improved LPS-induced HPMEC dysfunction and inflammatory response through SIRT1-mediated NF-κB deacetylation *in vitro*, which was consistent to this study.

As KEGG enriched analysis annotated, MAPK signaling pathway may take part in the effect of FGF21 on lung inflammation and hyperpermeability. The MAPK family mainly consists of Erk1/2 MAPK, JNK MAPK and P38 MAPK [35]. Our data suggested that FGF21 knockout upregulated the phosphorylation of JNK in murine lung while the phosphorylation of Erk1/2 and P38 showed no significant alterations. JNK MAPK signaling pathway is reported to exert biological functions such as endothelial inflammation [25, 36] and cellular tight junction (Q. [20–22]). The findings from the immunoblot analysis indicated that FGF21 suppressed the activation of JNK triggered by LPS. The study by Kang *et al.* demonstrated that the transplantation of FGF21-secreting adipose-derived stem cells suppressed the phosphorylation of JNK in the liver, aligning with our findings [37]. Nevertheless, the precise mechanisms underlying the relationship between FGF21 and JNK remain inadequately understood, necessitating further comprehensive investigation.

Previous studies reported that occludin degradation activated by the E3 ubiquitin ligase Itch is a critically regulatory mechanism in lung [38]. Itch was considered as a substrate of JNK [39]. JNK possibly facilitates occludin degradation via the E3 ubiquitin ligase Itch, which will need comprehensive exploration in the future. Our data showed that FGF21 inhibited the phosphorylation of JNK induced by LPS and rescued the downregulated occludin caused by LPS at the same time, which indicated that FGF21 possibly increased the occludin expression via inhibiting E3 ubiquitin ligase Itch by suppressing the activation of JNK.

For another, murine liver developed accumulated storage of fat when FGF21 is lacking in this investigation. Liver is a major metabolic organ for glucose and lipid metabolism and toxic metabolites degradation. In this study, we speculated that FGF21 deletion induced over accumulation of lipids in liver by metabolic perturbance of glucose and lipids. It is well-known that FGF21 is able to regulate metabolism via maintaining glucose tolerance and insulin sensitivity [40, 41]. A previous investigation determined that lipid accumulation exacerbates liver aging, while FGF21 enhances lipophagy in the liver to decrease lipid accumulation [42]. FGF21 also has been shown to inhibit hepatocyte senescence through the activation of the AMPK-dependent autophagic pathway [42] and by regulating macrophage polarization [43].

In skeletal muscle FGF21 is sufficient to activate muscle atrophy by activating the removal of damaged mitochondria through Bnip3-dependent mitophagy [44], which possesses anti-inflammatory effects [44]. Ablation of the mitophagy mediator PINK1 has been shown to induce lipid accumulation and trigger liver steatosis in preclinical models [45], which could be a strategy for balancing metabolic homeostasis, protecting or halting NAFLD. It is suggested that FGF21 may work coordinately with autophagy to regulate energy homeostasis in the context of aging [46]. Therefore, the effect of FGF21 on inflammation and metabolism during aging may be closely related to autophagy.

As for glucose metabolism, there is much dynamic balance between glucose and lipid metabolism to support energy balance in body. Surprisingly, the level of hepatic glucose showed no significant difference between FGF21 KO mice and widetype mice in this test, seemingly paradox to FGF21’s glucose-downregulating effect. Previous study indicated that 24-week-old male global FGF21-KO mice are insulin-resistant [47]. In this study, fructose 6-phosphate and glucose 6-phosphate, which are the key metabolites in glycolysis, are downregulated in FGF21-KO liver. This may be caused by the decrease of HK kinase activity in insulin resistance [48]. FGF21 knockout should have shown insulin resistance and elevated blood sugar in mice, but the active inflammatory processes also are in demand of high energy consumption, which result in no significant increase of glucose in FGF21 liver. Further regulatory mechanism on glucose and lipid metabolism resulted from genetic deletion of FGF21 is needed.

Although the perturbance of glucose and lipid metabolism is the predominant histological character of all types of liver disease, other less abundant metabolites are also key cellular factors in the development and progression of fatty liver. Firstly, higher level of aminoadipic acid [49] and 2-methylbutyroylcarnitine [50, 51] were highly predictive of patients who later progress to metabolic disorder. Especially, aminoadipic acid reduces fasting plasma glucose levels and increases insulin secretion from human islets. This study indicated that aminoadipic acid levels are elevated in liver of FGF21 KO mice, also supporting the hypothesis that increased insulin secretion and insulin resistance exist in FGF21 KO mice. In addition, a clinical study has been reported that compared with healthy individuals, patients with NASH and NAFLD have significantly higher levels of ceramides and other sphingolipid in liver, as well as the result that the ceramide levels are positively correlated with the extent of inflammation and oxidative stress [52, 53]. Analogously, Theodore *et al*. conducted a single-cell metabolomic analysis on the metabolic condition of human hepatocytes lines stimulated by fatty acids, and found that ceramides were highly concentrated in inflammatory steatosis hepatocytes. Those results were validated *in vivo* and consistent with previous reports [54]. Consistent with above studies, ceramide level is increased in FGF21 KO mice when compared with wide type mice in our result, as well as accumulation of fatty acids and TAG and increased cytokines in serum.

Enriched pathway analysis based on those significantly different metabolites were carried out further to explore the specific pathway influenced by FGF21. The glycerophospholipids metabolism, regarded as one of the metabolic pathways to characterize liver injury [55], is the top enriched pathway in current investigation. The disturbance of glycerophospholipids metabolism could impair hepatic metabolic function, cause steatohepatitis and metabolic syndrome, and finally develop liver fibrosis [56]. The excessive accumulation of LPC in hepatocytes has the potential to induce lipid toxicity, ultimately impairing hepatocyte metabolism [57, 58]. Previous study suggested that LPCAT3, the predominant LPCAT in the liver [59], plays a crucial role in LPC conversion, insulin-sensitivity regulation and systemic metabolism [59, 60]. The finding that loss of LPCAT3 has been shown to induce FGF21 secretion indirectly highlights a potential connection between FGF21 and LPCAT3 [60]. In this study, murine liver lacking FGF21 showed lower LPCAT3, seemly accounting for more hepatic accumulation of LPC. Considering that Glycerophospholipids metabolism is regulated by diversity of enzymes and complicated metabolic pathway, feedback regulation between FGF21 and LPCAT3 are possibly present.

## Conclusion

Aging mice lacking FGF21 spontaneously develop fatty liver and inflammatory response of lung under healthy feeding environment, indicating the critical role of FGF21 in aging-related metabolic disorder and inflammation. Multi-omics and further study reveal that FGF21 protects endothelial barrier function via enhancing occludin expression in LPS-induced pulmonary inflammation. And FGF21 regulate glucose and lipids metabolism and glycerophospholipid metabolism in aging-related fatty liver, which shed a new light into the pathophysiological role of FGF21 in aging-related diseases. This study enriches the knowledge of influence of FGF21 on aging process.

## Material and Method

### Animal Model

4–6 weeks C57BL/6 J male mice weighted at 20-25 g were all purchased from Vital River Laboratory Animal Technology. FGF21 KO mice backgrounded at C57BL/6 J were presented as a gift from Dr. Steve Kliewer (University of Texas Southwestern Medical Center, Dallas, TX, USA). FGF21 KO and WT littermates used in this study were bred from heterozygous mice. All mice were housed at 20–24 *°*C and fed with normal balanced diet.

LPS exposure was intratracheally injected with dose of 10 mg/kg LPS, while those in the control group were injected with an equal volume of PBS. Human recombinant FGF21 freeze-dried powder (97.9% purity and 124% activity) (D. [20–22, 61, 62]) comes from School of Pharmacy, Wenzhou Medical University. All experimental protocols were approved by the Institutional Animal Care and Use Committee of the Laboratory Animal Center at Wenzhou medical University.

### Measurement of Inflammatory Cytokines by ELISA

The perivascular blood sample was collected by removing eyeballs. After centrifugation, the supernatant was collected. The serum levels of IL-6, IL-1β, TNF-α, ICAM-1, VCAM-1 and TGF-β were measured using mouse ELISA kit (Shanghai Boyun Biotech, Co., Ltd., Shanghai, China). All serum samples were stored at − 80 *°*C until experiment and the procedures were performed following the manufacturer’s instructions. Each sample was analyzed in triplicate wells.

### Bronchoalveolar Lavage Fluid (BALF) Analysis

By lavaging the left lung four times repeatedly with 0.2 mL PBS each time, bronchoalveolar lavage fluid (BALF) was collected. Every lavaging was collected and recovery rate was kept above 80%. After centrifuged, the protein concentration was measured by Coomassie brilliant blue Assay and cell counts of BALF were analyzed by automated cell counter.

### Wet/Dry Ratio Analysis

After removing the lungs, the wet weight of the right lung from all groups was measured at once. Then all lungs were put in oven at 65 *°*C for 7 days to get dry wight measurement. Pulmonary edema was calculated as the ratio of Wet-to-Dry weight.

### Haematoxylin and Eosin (HE) Staining

Tissue slices from lungs were fixed in 4% paraformaldehyde overnight and embedded in paraffin. Subsequently they were cut into 5 μm using microtome, then deparaffinized, rehydrated and finally stained with haematoxylin and eosin. Images were acquired with a light microscope. The score was based on lung inflammatory condition. Scoring in each picture was based on the percentage of inflammatory area according to the following standard: 0 = no inflammation, 1 = up to 25%, 2 = 25%–50%, 3 = 50%–75%, 4 = 75%–100%.

### Immunohistochemistry

Firstly, the lung sections were were cut into 5 μm using microtome, deparaffinized and rehydrated. With incubation at 3% H_2_O_2_ at 37 *°*C for 10 min, the endogenous peroxidase activity was eliminated. The lung sections were boiled in antigen retrieval buffer containing citrate-hydrochloric acid for 15 min and blocked with 5% normal goat serum (OriGENE Technologies, Inc.) for 30 min. Then the sections were incubated with anti-MPO antibody (22225-1-AP, proteintech) or anti-CD68 antibody (28058-1-AP, proteintech) overnight at 4 *°*C. The next day, the sections were incubated with biotin-labeled secondary antibody working solution (1:200, A0277, Beyotime, Shanghai, China; Goat anti-rabbit IgG-HRP) at 37 *°*C for 30 min. The morphology of lung slides was scanned with Nikon inverted microscope (Nikon, Tokyo, Japan).

### Evans Blue Permeable Assessment and FITC-dextran Permeable Assessment

One hour before sacrifice, FITC-dextran (70 kDa; 10 mg/kg, Sigma-Aldrich) or Evans blue dye (25 mg/kg, BBI solutions) was injected intravenously via tail vein. Lungs were perfused through cardiac lavage with PBS and were put in oven at 60 *°*C for 24 h. Lung tissues were weighed and soaked in methanamide (0.03 mL/m) for 24 h at 60 *°*C and then centrifuged. Absorbance at 620 nm and 740 nm of the supernatant was measured. Adjustment was calculated on the following formula: corrected absorbance at 620 nm = actual absorbance at 620 nm − [1.426 × (absorbance at 740 nm) + 0.03].

After FITC-dextran injection for 1 h, lung tissues were perfusing and frozen by liquid nitrogen. After cut into 8 μm, the leaking FITC-dextran was recorded by Confocal laser scanning microscopy (Nikon, Tokyo, Japan).

### Immunofluorescence Detection

The lung immunofluorescence frozen sections were used for immunofluorescence after fixed by 4% formaldehyde. Then slides were blocked with 5% donkey serum one hour at room temperature and incubated with anti-occludin antibody (1:100, Huabio, R1510-33) overnight at 4 *°*C. After incubated with secondary antibodies anti-rabbit IgG-594 (1:100, abcam, ab150076) And nuclei were stained with 4,6-diamidino-2-phenylindole (DAPI). Fluorescence images were recorded by confocal laser scanning microscopy (Nikon, Tokyo, Japan).

### PCR Array

Total RNA extraction was performed with Trizol reagent (Takara). OD260/OD280 ratio greater than 1.8 were set for standard sample selection. Then cDNA was prepared according to iScript cDNA Synthesis kit (Bio-Rad, USA) following the manufacturer’s instructions. Then cDNA was added into a mouse PCR arrary plate (Wcgene Biotech, Shanghai, China) to detect gene expression profiling. The qPCR was performed using the Real-Time PCR System (CFX96 Real-Time System, Bio-Rad, USA), and the total experiment was performed according to the manufacturer's protocol. Data were normalized to the gene expression based on the cycle threshold (CT) values and were used to calculate relative gene expression with the 2^−ΔΔCT^ method. The significantly different genes were selected with fold regulation greater than 2 and *P*-value < 0.05.

### RNA-Sequencing

Total RNA from the lung was obtained for RNA-sequencing library construction and following sequencing. Then a comprehensive transcriptome from all samples were obtained from paired-end sequencing, which was performed on an Illumina Hiseq X-Ten (LC Bio, China) and analyzed by perl scripts. After the final transcriptome was generated, FPKM values (fragments per kilobase of exon per million fragments mapped) by the Cufflinks software was applied to evaluate gene expression levels. The P-value was identified by the FDR (false discovery rate) control method and the differentially-expressed messenger RNAs (mRNAs) were selected with P-value < 0.05 and |log2 (fold change)|> 1, all above was performed by R software.

### Cell Culture

Human umbilical vascular endothelial cell (HUVEC) was purchased from ATCC and cultured in an atmosphere of 5% CO2 at 37 °C. Overexpression of FGF21 in HUVEC was carried out by lentiviral infection following the manufacturer’s protocol. Lentiviral vectors with FGF21-green fluorescent protein (GFP) genes (Lv-FGF21) or without FGF21 genes (Lv-CON) were constructed by the Ji Kai Gene Company (Shanghai, China). 3 days after the lentivirus infection, fresh medium containing 1 μg/mL puromycin was added to select stable puromycin-resistant cells. Overexpression efficiency was testified by western blot and PCR. Cells in the corresponding group were treated with LPS at a concentration of 0.5 mg/mL. Activation of JNK MAPK signaling pathway was applied by Anisomycin with dose of 1 μg/mL (Aladdin, A102397).

### Real-Time qPCR

Trizol reagent (Takara) was used to extract total RNA from cells. And only samples with an OD260/OD280 ratio greater than 1.8 were used for experiments. The cDNA was prepared according to iScript cDNA Synthesis kit (Bio-Rad, USA) according to the manufacturer’s instructions. And qPCR was performed using the Real-Time PCR System (CFX96 Real-Time System, Bio-Rad, USA). β-actin served as endogenous control. The relative fold change was calculated using the 2^–△△CT^ method. Forward primer ATGGATCGCTCCACTTTGACC and reverse primer GGGCTTCGGACTGGTAAACAT for FGF21. Forward primer CTGGAACGGTGAAGGTGACA and reverse primer AAGGGACTTCCTGTAACAATGCA for β-actin.

### Western Blot

Cell and tissue lysates were homogenized and mixed with loading buffer. Samples were then boiled for 10 min. Separate samples were loaded onto a 10% SDS-PAGE gel. Separated proteins were transferred to PVDF (Millipore, Billerica, MA, USA). The membranes were blocked with 5% nonfat milk at room temperature for 1.5 h and incubated with specific primary antibodies overnight at 4°C. All primary antibodies concentration were diluted 1000 times. After incubation with secondary antibodies, the stripes were treated with ECL reagent (Pierce, WI, USA) for detection. The protein signals were quantified with Image J software after visualized by Bio-Rad Electrochemiluminescence detector. The primary antibodies used were as follows: rabbit anti-occludin (1:1000, R1510-33, HUABIO), rabbit anti-ZO-1 (1:1000, ab276131, Abcam), rabbit anti-claudin5 (1:1000, ET1703-58, HUABIO), rabbit anti-E-cadherin (1:1000, #3195, CST), rabbit anti-phospho-JNK (1:1000, ET1609-42, HUABIO), rabbit anti-JNK (1:1000, ET1601-28, HUABIO), rabbit anti-FGF21 (1:1000, ab171941, Abcam), rabbit anti-phospho-Erk (1:1000, #4370, CST), rabbit anti-Erk (1:1000, #4695, CST), rabbit anti-phospho-P38 (1:1000, ab195049, Abcam) and rabbit anti-P38 (1:1000, ab170099, Abcam), mouse anti-LPCAT3 (1:1000, 678821-Ig, proteintech), rabbit anti-β-tubulin (1:1000, #2128, CST) and rabbit anti-β-actin (1:1000, 81115-1-RR, proteintech). β-tubulin or β-actin was used as an internal control.

### Measurement of Monolayer Permeability

For endothelial monolayer permeability evaluation, HUVEC (5 × 10^3^ cells/well) transfected with Lv-FGF21 or Lv-CON were seeded on polyethylene terephthalate transwell filters (Corning Costar 3470; Corning Inc.). After reaching confluence, LPS was administrated for 24 h (0.5 mg/mL, Sigma-Aldrich) and then FITC-dextran (10 mg/mL, Sigma-Aldrich) was added to the top well for 1 h. The fluorescence intensity of leaking FITC-dextran in the lower room was measured by multifunctional microplate reader (TECAN, Maennedorf, Switzerland) at 488 nm excitation and 520 nm emission.

### Metabolomics Analysis and Lipidomics Analysis

The liver samples were harvested and underwent targeted metabolomics profiling analysis. Firstly, Q300 Kit (Metabo-Profile, Shanghai, China) was used to generate all raw metabolites data by UPLC-MS/MS. Then data was processed at iMAP platform. For another, lipid profiling of liver was also generated on a Waters ACQUITY Ultra-Performance LC (UPLC) system. A Waters XEVO TQ-S mass spectrometry and MassLynx 4.1 software (Waters, Milford, MA) were also used to generate and process data. At first, distribution characteristics of metabolite profile between groups was identified by Principal component analysis (PCA) and orthogonal partial least squares discriminant analysis. According to the OPLS-DA model, VIP (variable importance in projection) was obtained and was used for following process. Metabolites with p-value < 0.05 and VIP ≥ 1 were screened as statistically significant metabolites. Following analysis of enriched pathway was performed with selected metabolites.

### Statistics

ImageJ (NIH) software was used to analyze protein bands gray scale scanning. GraphPad Prism 7.0 (GraphPad Software, San Diego, CA, USA) was applied to perform statistical analyses. Comparisons between two groups were analyzed by unpaired two-tailed Student's t-test and multiple comparisons were analyzed by ANOVA followed by Bonferroni post hoc test. *P* < *0.05* was indicated as significance. All data were from at least three independent experiments and presented as the mean ± SD.

## Supplementary Information

Below is the link to the electronic supplementary material.

Supplementary Fig. 1  FGF21 knockout showed no histomorphologic change and perivascular inflammatory alteration in 4–6 weeks mice. **a** Representative H&E‐stained lung, liver, and spleen sections in 4–6 weeks mice of FGF21 KO mice and WT mice. ×200, Scale bar, 100 μm (n = 7). **b** The cytokines concentrations in serum of mice aged at 4–6 weeks (n = 7; ^*^P < 0.05, ^**^P < 0.01 vs. Y-WT mice). **c** The heatmap summarize the mRNA level of inflammatory cytokines and related receptors in the spleen of O-FGF21 KO mice and O-WT mice. Gene expression levels were normalized as Log2(fold change in O-FGF21 KO mice vs O-WT mice) (blue: downregulated, red: upregulated, white: medium change) (n = 3) (TIFF 32 KB)

Supplementary Fig. 2 Supplementary result of Omics analysis.**a** heatmap of differential genes between Y-FGF21-KO+LPS mice and Y-WT+LPS mice. **b** KEGG enriched analysis from differential genes between Y-FGF21-KO+LPS mice and Y-WT+LPS mice. **c** PCA (Principal component analysis) shows the distribution feature of metabolites between O-FGF21-KO+LPS mice and O-WT+LPS mice (TIFF 32 KB)

Supplementary Fig. 3 Representative metabolites in Omics analysis (TIFF 32 KB)

Supplementary file4 (DOCX 26 KB)

## Data Availability

No datasets were generated or analysed during the current study.
