# Supplementary figures and images for: Investigation of Metabolic and Inflammatory Disorder in the Aging FGF21 Knockout Mouse
Source: Inflammation. 2024 Apr 24;47(6):2173–95. doi: 10.1007/s10753-024-02032-3 (PMC11607023; doi:10.1007/s10753-024-02032-3)

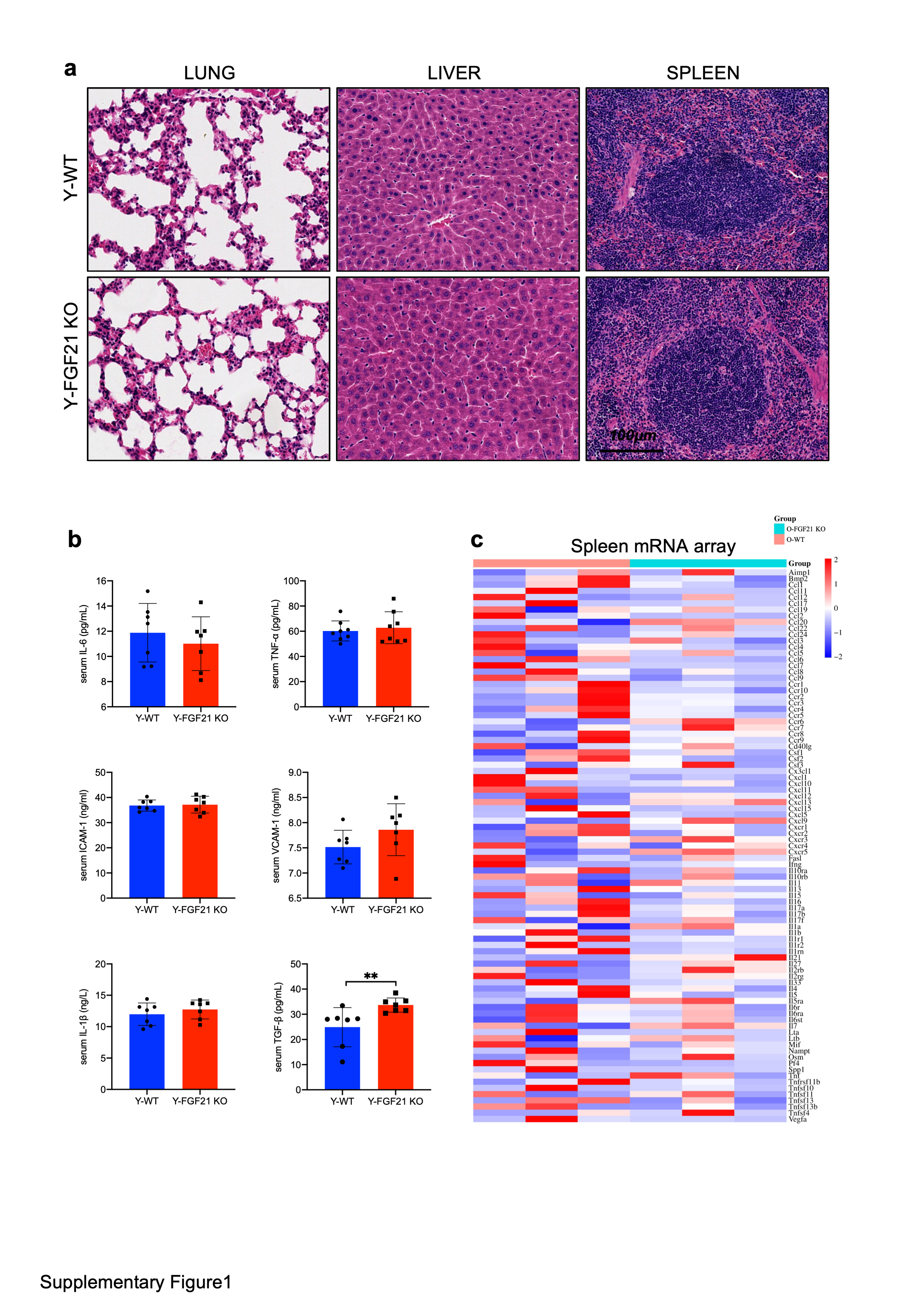

Supplement: Supplementary file 1 — Supplementary Fig. 1 FGF21 knockout showed no histomorphologic change and perivascular inflammatory alteration in 4–6 weeks mice. a Representative H&E‐stained lung, liver, and spleen sections in 4–6 weeks mice of FGF21 KO mice and WT mice. ×200, Scale bar, 100 μm (n = 7). b The cytokines concentrations in serum of mice aged at 4–6 weeks (n = 7; *P < 0.05, **P < 0.01 vs. Y-WT mice). c The heatmap summarize the mRNA level of inflammatory cytokines and related receptors in the spleen of O-FGF21 KO mice and O-WT mice. Gene expression levels were normalized as Log2(fold change in O-FGF21 KO mice vs O-WT mice) (blue: downregulated, red: upregulated, white: medium change) (n = 3) (TIFF 32 KB) [file 10753_2024_2032_MOESM1_ESM.tiff]

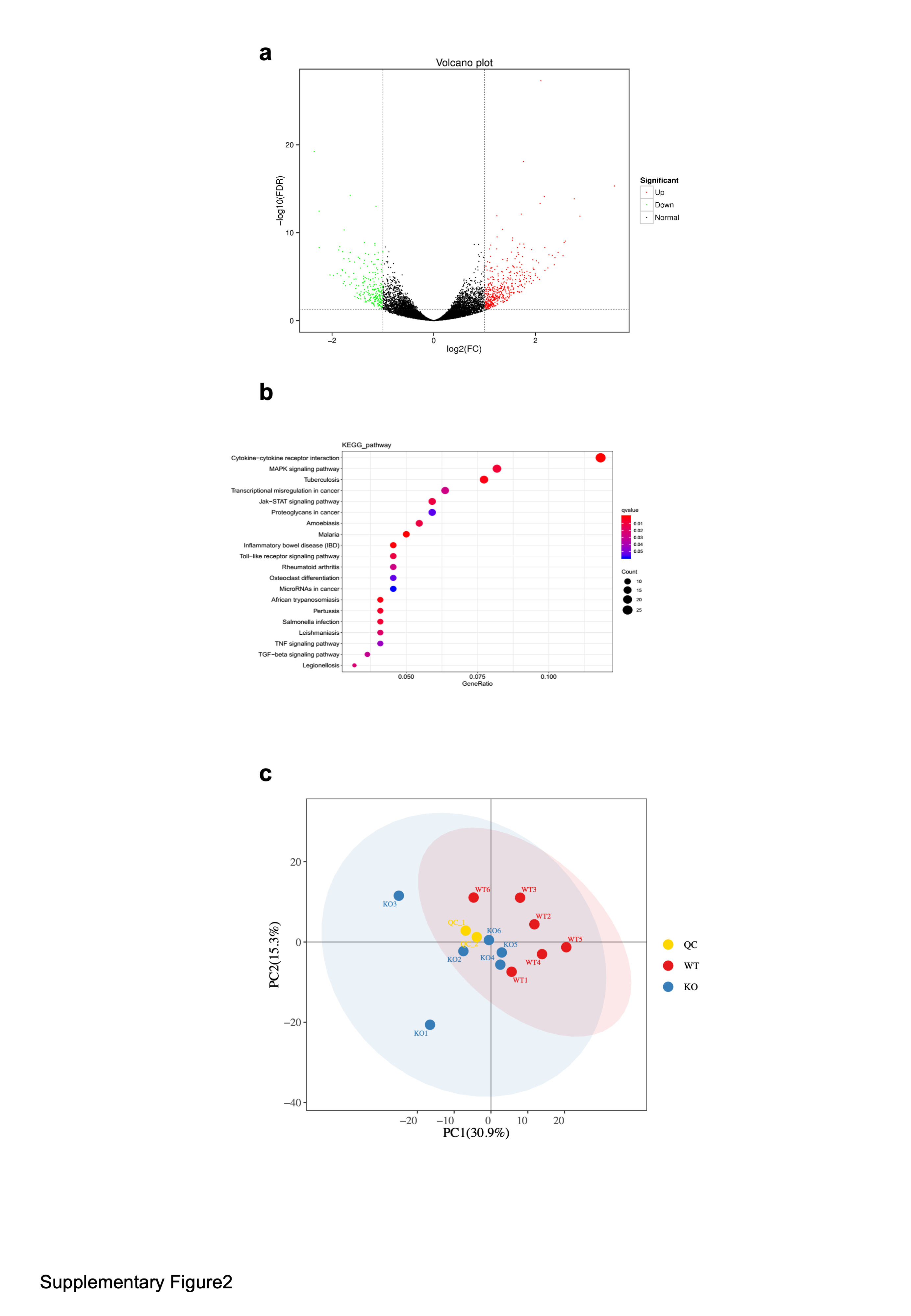

Supplement: Supplementary file 2 — Supplementary Fig. 2 Supplementary result of Omics analysis.a heatmap of differential genes between Y-FGF21-KO+LPS mice and Y-WT+LPS mice. b KEGG enriched analysis from differential genes between Y-FGF21-KO+LPS mice and Y-WT+LPS mice. c PCA (Principal component analysis) shows the distribution feature of metabolites between O-FGF21-KO+LPS mice and O-WT+LPS mice (TIFF 32 KB) [file 10753_2024_2032_MOESM2_ESM.tiff]

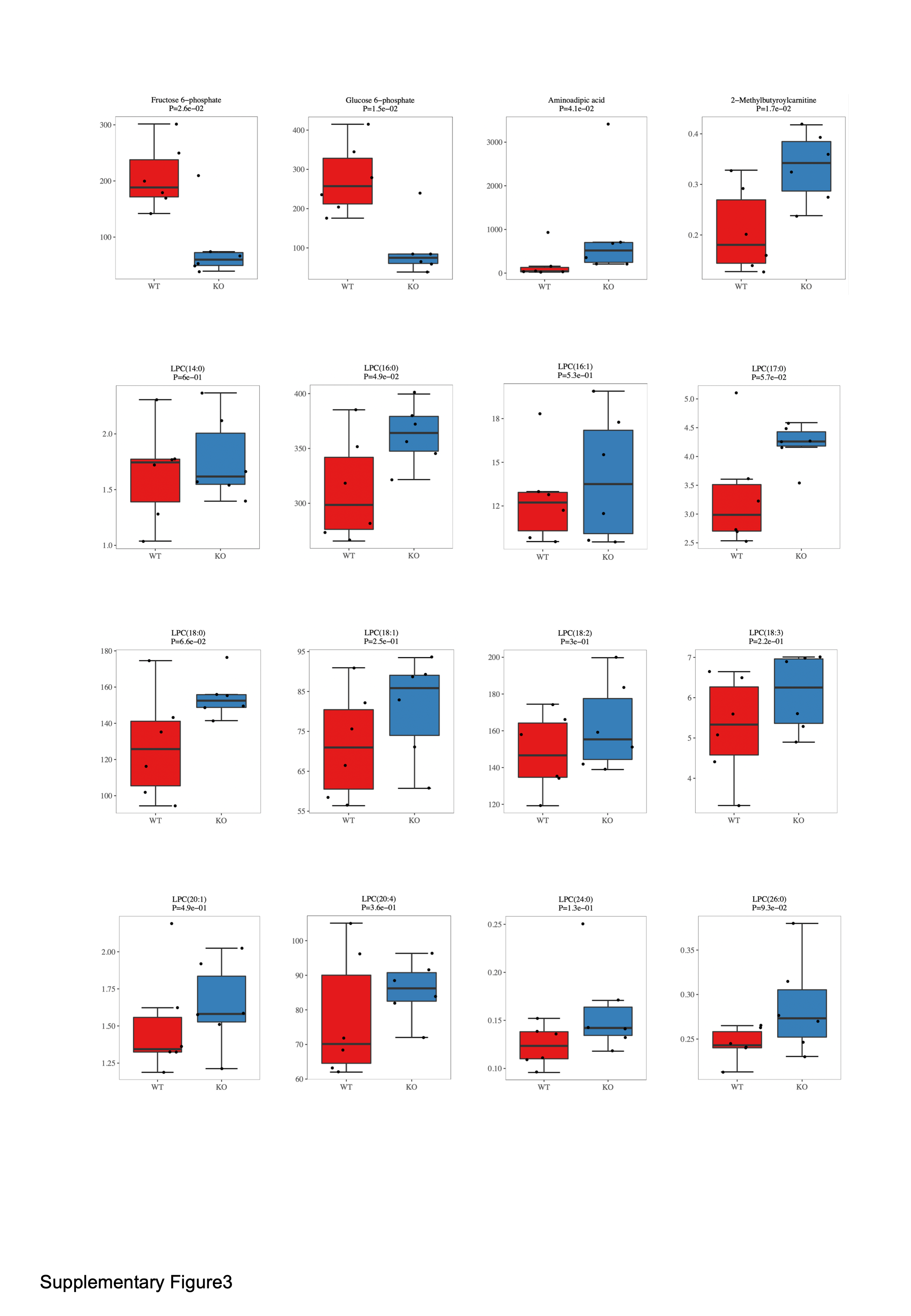

Supplement: Supplementary file 3 — Supplementary Fig. 3 Representative metabolites in Omics analysis (TIFF 32 KB) [file 10753_2024_2032_MOESM3_ESM.tiff]
