# Supplementary material for: Investigation of Metabolic and Inflammatory Disorder in the Aging FGF21 Knockout Mouse
Source: Inflammation. 2024 Apr 24;47(6):2173–95. doi: 10.1007/s10753-024-02032-3 (PMC11607023; doi:10.1007/s10753-024-02032-3)
Supplement: Supplementary file 4 — Supplementary file4 (DOCX 26 KB) [file 10753_2024_2032_MOESM4_ESM.docx]

**Supplementary Table1** **122 differential metabolites identified in liver samples between the FGF21 KO mice and WT mice are presented stratified by group.**

| Metabolites | log_2_ (FC) | Ρ-value | Uni_FDR | OPLSDA_VIP |
| --- | --- | --- | --- | --- |
| **Amino Acids** |  |  |  |  |
| Glutamic acid | -0.39 | 1.30E-02 | 1.26E-01 | 1.54 |
| Homoserine | 1.90 | 1.42E-02 | 1.26E-01 | 1.41 |
| 2-Phenylglycine | 0.75 | 5.00E-02 | 1.89E-01 | 1.30 |
| Aminoadipic acid | 3.68 | 4.11E-02 | 1.76E-01 | 1.17 |
| alpha-Aminobutyric acid | 0.52 | 2.66E-03 | 1.14E-01 | 1.76 |
| Pipecolic acid | 0.32 | 1.95E-02 | 1.29E-01 | 1.42 |
| Pyroglutamic acid | -0.44 | 1.86E-02 | 1.29E-01 | 1.43 |
| 4-Hydroxyproline | 0.57 | 1.97E-02 | 1.29E-01 | 1.65 |
|  |  |  |  |  |
| **Carbohydrates** |  |  |  |  |
| Ribonic acid | 1.04 | 2.13E-03 | 1.14E-01 | 1.62 |
| Maltotriose | -1.21 | 2.16E-03 | 1.14E-01 | 1.76 |
| Fructose 6-phosphate | -1.66 | 2.60E-02 | 1.38E-01 | 1.67 |
| Glucose 6-phosphate | -1.78 | 1.52E-02 | 1.26E-01 | 1.74 |
|  |  |  |  |  |
| **Carnitines** |  |  |  |  |
| Acetylcarnitine | 0.88 | 4.33E-03 | 1.14E-01 | 1.45 |
| 2-Methylbutyroylcarnitine | 0.69 | 1.72E-02 | 1.26E-01 | 1.46 |
| Valerylcarnitine | 1.60 | 2.16E-03 | 1.14E-01 | 1.50 |
| 3-Hydroxylisovalerylcarnitine | 0.29 | 4.36E-02 | 1.79E-01 | 1.31 |
| Glutarylcarnitine | 0.92 | 2.43E-03 | 1.14E-01 | 1.60 |
|  |  |  |  |  |
| **Cer** |  |  |  |  |
| Cer(d18:1/18:0) | 0.76 | 3.06E-02 | 1.52E-01 | 1.27 |
| Cer(d18:1/23:0) | 0.89 | 4.11E-02 | 1.76E-01 | 1.41 |
| Cer(d18:1/24:0) | 0.54 | 2.60E-02 | 1.38E-01 | 1.33 |
| Cer(d18:1/24:1) | 0.95 | 8.66E-03 | 1.24E-01 | 1.62 |
| Cer(d18:2/20:0) | 0.91 | 2.20E-02 | 1.35E-01 | 1.29 |
| Cer(d18:2/23:1) | 1.04 | 4.33E-03 | 1.14E-01 | 1.46 |
|  |  |  |  |  |
| **Fatty Acids** |  |  |  |  |
| Azelaic acid | 1.19 | 4.11E-03 | 1.14E-01 | 1.55 |
| Sebacic acid | 0.77 | 1.12E-02 | 1.26E-01 | 1.60 |
| Methylsuccinic acid | 0.93 | 1.52E-02 | 1.26E-01 | 1.60 |
| Methylglutaric acid | 0.46 | 4.11E-02 | 1.76E-01 | 1.22 |
| Pimelic acid | 0.78 | 3.51E-02 | 1.63E-01 | 1.26 |
| 3-Methyladipic acid | 1.50 | 8.66E-03 | 1.24E-01 | 1.61 |
| Nonanoic acid | 0.25 | 2.15E-02 | 1.35E-01 | 1.47 |
| Dihomo-gamma-linolenic acid | -0.64 | 1.69E-02 | 1.26E-01 | 1.65 |
| 2,2-Dimethylsuccinic acid | 0.41 | 3.51E-02 | 1.63E-01 | 1.44 |
|  |  |  |  |  |
| **Nucleotides** |  |  |  |  |
| SAH | 0.66 | 4.98E-04 | 1.14E-01 | 1.84 |
| AMP | 1.56 | 2.16E-03 | 1.14E-01 | 1.42 |
| GMP | 3.13 | 8.66E-03 | 1.24E-01 | 1.43 |
|  |  |  |  |  |
| **Organic Acids** |  |  |  |  |
| Hydroxypropionic acid | 0.65 | 1.27E-02 | 1.26E-01 | 1.67 |
| Glutaric acid | 0.94 | 2.60E-02 | 1.38E-01 | 1.61 |
| 2-Hydroxybutyric acid | 1.09 | 3.07E-03 | 1.14E-01 | 1.87 |
| Glutaconic acid | 0.41 | 4.91E-02 | 1.89E-01 | 1.31 |
| Malonic acid | 0.34 | 7.35E-03 | 1.24E-01 | 1.54 |
| Oxoadipic acid | 2.34 | 2.60E-02 | 1.38E-01 | 1.41 |
|  |  |  |  |  |
| **PC** |  |  |  |  |
| PC(24:0) | 0.42 | 1.52E-02 | 1.26E-01 | 1.59 |
| PC(30:0) | 0.49 | 5.33E-03 | 1.24E-01 | 1.56 |
| PC(32:0) | 0.33 | 1.89E-02 | 1.29E-01 | 1.41 |
| PC(32:1) | 0.31 | 3.52E-02 | 1.63E-01 | 1.18 |
| PC(34:0) | 0.61 | 1.09E-02 | 1.26E-01 | 1.52 |
| PC(36:0) | 0.64 | 1.52E-02 | 1.26E-01 | 1.78 |
| PC(38:0) | 0.74 | 2.60E-02 | 1.38E-01 | 1.57 |
| PC(38:1) | 0.80 | 2.81E-02 | 1.42E-01 | 1.40 |
| PC(40:1) | 0.94 | 3.19E-02 | 1.55E-01 | 1.44 |
| PC(40:4) | 0.40 | 2.60E-02 | 1.38E-01 | 1.11 |
| PC(40:7) | 0.44 | 8.66E-03 | 1.24E-01 | 1.71 |
| PC(40:8) | 0.55 | 4.33E-03 | 1.14E-01 | 1.65 |
| PC(42:0) | 1.02 | 1.03E-02 | 1.26E-01 | 1.34 |
| PC(42:10) | 0.65 | 1.27E-02 | 1.26E-01 | 1.49 |
| PC(42:3) | 0.66 | 4.39E-02 | 1.79E-01 | 1.37 |
| PC(42:4) | 0.62 | 4.60E-02 | 1.86E-01 | 1.37 |
| PC(42:6) | 0.45 | 1.52E-02 | 1.26E-01 | 1.59 |
| PC(42:8) | 0.38 | 1.73E-02 | 1.26E-01 | 1.55 |
| PC(42:9) | 0.45 | 2.15E-02 | 1.35E-01 | 1.48 |
| PC(O-30:0) | 0.33 | 4.98E-02 | 1.89E-01 | 1.25 |
| PC(O-32:0) | 0.35 | 1.74E-02 | 1.26E-01 | 1.47 |
| PC(O-34:0) | 0.58 | 1.66E-03 | 1.14E-01 | 1.73 |
| PC(O-34:1) | 0.52 | 5.99E-03 | 1.24E-01 | 1.57 |
| PC(O-36:4) | 0.41 | 2.67E-02 | 1.39E-01 | 1.31 |
| PC(O-38:5) | 0.50 | 5.67E-03 | 1.24E-01 | 1.56 |
| PC(O-38:6) | 0.51 | 1.74E-02 | 1.26E-01 | 1.30 |
| PC(O-40:1) | 1.03 | 4.90E-02 | 1.89E-01 | 1.25 |
| PC(O-40:4) | 0.83 | 2.58E-02 | 1.38E-01 | 1.33 |
| PC(O-40:5) | 0.29 | 1.52E-02 | 1.26E-01 | 1.19 |
|  |  |  |  |  |
| **PE** |  |  |  |  |
| ePE(34:2) | 1.02 | 5.92E-03 | 1.24E-01 | 1.47 |
| ePE(40:6) | 0.74 | 4.85E-02 | 1.89E-01 | 1.39 |
| PE(32:1) | 0.73 | 1.90E-02 | 1.29E-01 | 1.31 |
| PE(32:2) | 0.74 | 1.42E-02 | 1.26E-01 | 1.34 |
| PE(34:1) | 0.90 | 1.76E-02 | 1.26E-01 | 1.43 |
| PE(34:2) | 0.62 | 4.17E-02 | 1.76E-01 | 1.34 |
| PE(34:3) | 0.52 | 3.08E-02 | 1.52E-01 | 1.23 |
| PE(34:4) | 0.68 | 1.67E-02 | 1.26E-01 | 1.48 |
| PE(36:0) | 0.29 | 1.52E-02 | 1.26E-01 | 1.08 |
| PE(36:1) | 0.67 | 4.11E-02 | 1.76E-01 | 1.20 |
| PE(36:2) | 0.75 | 4.88E-02 | 1.89E-01 | 1.37 |
| PE(36:3) | 0.63 | 1.17E-02 | 1.26E-01 | 1.53 |
| PE(36:4) | 0.57 | 1.32E-02 | 1.26E-01 | 1.49 |
| PE(38:3) | 0.72 | 4.06E-02 | 1.76E-01 | 1.40 |
| PE(38:4) | 0.65 | 2.60E-02 | 1.38E-01 | 1.51 |
| PE(38:5) | 0.67 | 8.48E-03 | 1.24E-01 | 1.52 |
| PE(38:6) | 0.55 | 1.05E-02 | 1.26E-01 | 1.60 |
| PE(40:3) | 0.85 | 2.60E-02 | 1.38E-01 | 1.09 |
| PE(40:6) | 0.71 | 4.25E-02 | 1.78E-01 | 1.41 |
| PE(40:7) | 0.66 | 6.42E-03 | 1.24E-01 | 1.61 |
| PE(40:8) | 0.76 | 4.00E-03 | 1.14E-01 | 1.68 |
| PE(42:9) | 0.58 | 8.66E-03 | 1.24E-01 | 1.40 |
| PE(44:12) | 0.42 | 4.40E-02 | 1.79E-01 | 1.26 |
|  |  |  |  |  |
| **SM** |  |  |  |  |
| SM(d17:1/27:2) | 0.52 | 4.17E-02 | 1.76E-01 | 1.31 |
| SM(d18:0/17:0) | 0.36 | 1.41E-02 | 1.26E-01 | 1.43 |
| SM(d18:0/24:0) | 0.79 | 3.39E-02 | 1.63E-01 | 1.42 |
| SM(d18:0/26:2) | 0.85 | 2.23E-02 | 1.35E-01 | 1.45 |
| SM(d18:1/19:0) | 0.42 | 8.37E-03 | 1.24E-01 | 1.59 |
| SM(d18:2/23:1) | 0.41 | 2.02E-02 | 1.31E-01 | 1.37 |
|  |  |  |  |  |
| **TAG** |  |  |  |  |
| TG(46:2) | 0.25 | 2.60E-02 | 1.38E-01 | 1.49 |
| TG(48:0) | 0.37 | 6.59E-03 | 1.24E-01 | 1.73 |
| TG(51:3) | 0.31 | 1.52E-02 | 1.26E-01 | 1.74 |
| TG(52:2) | 0.30 | 4.33E-03 | 1.14E-01 | 1.73 |
| TG(52:3) | 0.29 | 1.52E-02 | 1.26E-01 | 1.64 |
| TG(52:4) | 0.29 | 2.58E-02 | 1.38E-01 | 1.52 |
| TG(52:6) | 0.29 | 2.80E-02 | 1.42E-01 | 1.49 |
| TG(54:2) | 0.27 | 4.08E-02 | 1.76E-01 | 1.46 |
| TG(54:3) | 0.26 | 2.69E-02 | 1.39E-01 | 1.50 |
| TG(54:4) | 0.29 | 8.66E-03 | 1.24E-01 | 1.66 |
| TG(54:5) | 0.30 | 1.57E-02 | 1.26E-01 | 1.60 |
| TG(54:6) | 0.30 | 1.75E-02 | 1.26E-01 | 1.56 |
| TG(54:7) | 0.30 | 2.71E-02 | 1.39E-01 | 1.46 |
| TG(56:4) | 0.52 | 2.46E-03 | 1.14E-01 | 1.64 |
|  |  |  |  |  |
| **Others** |  |  |  |  |
| LPC(16:0) | 0.22 | 4.95E-02 | 1.89E-01 | 1.19 |
| Indole-3-propionic acid | 0.87 | 1.01E-03 | 1.14E-01 | 1.81 |
| Imidazolepropionicacid | 0.43 | 4.17E-02 | 1.76E-01 | 1.46 |
| UDCA | 1.13 | 1.93E-02 | 1.29E-01 | 1.59 |
| Phthalic acid | 0.59 | 9.08E-04 | 1.14E-01 | 1.83 |
| Propionic acid | 0.35 | 1.63E-02 | 1.26E-01 | 1.69 |
| PS(38:5) | 0.69 | 1.52E-02 | 1.26E-01 | 1.50 |
| PI(40:4) | 0.77 | 3.47E-02 | 1.63E-01 | 1.32 |
| Phenyllactic acid | 0.87 | 2.23E-02 | 1.35E-01 | 1.35 |
